# Atomic-Scale Insights into Alloying-Induced Interfacial Stability, Adhesion, and Electronic Structure of Mg/Al_3_Y Interfaces

**DOI:** 10.3390/ma19030562

**Published:** 2026-01-30

**Authors:** Yunxuan Zhou, Liangjuan Gao, Quanhui Hou, Jun Tan, Zhao Ding

**Affiliations:** 1 Lanxi Magnesiun Materials Research Institute, Lanxi 321100, China; yunxuanzhou@cqu.edu.cn (Y.Z.); jun.tan@cqu.edu.cn (J.T.); 2 National Engineering Research Center for Magnesium Alloys, College of Materials Science and Engineering, Chongqing University, Chongqing 400044, China; 3 College of Materials Science and Engineering, Sichuan University, Chengdu 610065, China; lgao87@scu.edu.cn; 4 School of Automotive Engineering, Yancheng Institute of Technology, Yancheng 224051, China

**Keywords:** magnesium matrix composites, interfacial segregation behavior, doping elements, electronic properties, first-principles calculations

## Abstract

This work aims to enhance the stability of the Mg/Al_3_Y interface through first-principles investigations of low-cost dopants. Density functional theory calculations were employed to systematically examine the bulk properties of Mg and Al_3_Y, as well as the structural stability, electronic characteristics, and alloying element effects at the Mg(0001)/Al_3_Y(0001) interface. The calculated lattice parameters, elastic moduli, and phonon spectra demonstrate that both Mg and Al_3_Y are dynamically stable. Owing to the similar hexagonal symmetry and a small lattice mismatch (~1.27%), a low-strain semi-coherent Mg(0001)/(2 × 2)Al_3_Y(0001) interface can be constructed. Three representative interfacial stacking configurations (OT, MT, and HCP) were examined, among which the MT configuration exhibits significantly higher work of adhesion, indicating superior interfacial stability. Differential charge density and density of states analyses reveal pronounced charge transfer from Mg to Al/Y atoms and strong orbital hybridization, particularly involving Y-d states, which underpins the enhanced interfacial bonding. Furthermore, the segregation behavior and adhesion enhancement effects of typical alloying elements (Si, Ca, Ti, Mn, Cu, Zn, Zr, and Sn) were systematically evaluated. The results show that Mg-side interfacial sites, especially Mg_2_ and Mg_3_, are thermodynamically favored for segregation, with Zr and Ti exhibiting the strongest segregation tendency and the most significant improvement in interfacial adhesion. These findings provide fundamental insights into interfacial strengthening mechanisms and offer guidance for the alloy design of high-performance Mg-based composites.

## 1. Introduction

Magnesium alloys are among the lightest structural metallic materials and have attracted sustained interest for applications in aerospace, automotive lightweighting, and electronic industries owing to their low density, high specific strength, and excellent damping capacity [1,2,3,4]. However, the widespread application of conventional Mg alloys is still limited by their relatively low room-temperature strength, poor thermal stability, and inadequate creep resistance under elevated-temperature service conditions [5,6,7]. Alloying with rare-earth (RE) elements has proven to be an effective strategy to overcome these limitations [8,9]. RE additions can significantly refine grain structures, modify deformation textures, enhance thermal stability, and suppress grain-boundary-mediated deformation mechanisms [10,11]. As a result, Mg–RE-based alloys, particularly Mg–Al–RE systems, have emerged as promising candidates for next-generation lightweight structural materials. Nevertheless, the strengthening mechanisms induced by RE elements are intrinsically complex and are closely associated with the formation, stability, and interfacial behavior of RE-containing secondary phases, which remain insufficiently understood at the atomic scale [12,13].

In Mg–Al–RE alloys, RE elements tend to participate in the formation of thermodynamically stable intermetallic compounds, among which Al_3_RE phases play a critical role due to their high melting points, structural stability, and superior mechanical stiffness [14,15]. Experimental investigations on Mg–Al–RE and Al–RE systems have demonstrated that Al_3_RE phases (e.g., Al_3_Ce) can effectively enhance hardness, wear resistance, and high-temperature mechanical performance by acting as stable reinforcement phases [16]. Diffusion-couple studies have further revealed the formation kinetics and growth behavior of Al–RE intermetallic layers at Mg/Al–RE interfaces under various thermal conditions [17,18]. Complementary first-principles calculations have systematically evaluated the formation energies, electronic structures, elastic constants, and thermodynamic properties of a wide range of Al_3_RE compounds, confirming their intrinsic stability and strong bonding characteristics [14,19,20,21]. These studies collectively indicate that Al_3_RE phases are not only key strengthening constituents in Mg–RE alloys but also provide a robust platform for tailoring interfacial properties in multiphase Mg-based systems.

In multiphase alloys and metal–intermetallic composites, interfacial structure and properties play a decisive role in load transfer efficiency, deformation compatibility, and failure behavior [22,23]. Despite extensive investigations into the formation and bulk properties of Al_3_RE phases, most existing studies have focused on phase equilibria, microstructural evolution, and macroscopic mechanical responses, while the atomic-scale characteristics of Mg/Al_3_RE interfaces remain largely unexplored. Previous first-principles studies have predominantly addressed Al/Al_3_RE or fcc-Al/L1_2_-Al_3_M interfaces, elucidating interfacial energies, preferred orientation relationships, and adhesion strengths [24,25]. However, direct investigations of interfaces between hcp Mg and Al_3_RE intermetallics are notably scarce, despite their practical relevance in Mg–Al–RE alloys. Understanding the pronounced differences in crystal structure, electronic configuration, and bonding nature between Mg and Al_3_RE is therefore essential to clarify interfacial stability, adhesion strength, and electronic interactions. In this work, we focus on the Mg/Al_3_Y interface, as yttrium (Y) is an effective strengthening element for the Mg matrix, enhancing its mechanical properties [2,26], relatively inexpensive compared with other rare-earth elements [27], and readily forms the stable intermetallic compound Al_3_Y with aluminum [28,29]. Using first-principles calculations, we systematically investigate the interface’s structural stability, adhesion, and electronic properties. The results provide atomistic insights into interfacial bonding mechanisms and their correlation with mechanical response, offering theoretical guidance for interface engineering and microstructural optimization in high-performance Mg–Al–RE alloys.

## 2. Theoretical Section

First-principles simulations were conducted to elucidate the bulk, surface, and interfacial characteristics of the Mg/Al_3_Y system within the density functional theory framework, as implemented in the CASTEP code (Materials Studio 8.0) [30]. Element-specific valence states were explicitly considered in the construction of the pseudopotentials, with Mg, Al, and Y treated using 2p^6^3s^2^, 3s^2^3p^1^, and 4s^2^4p^6^4d^1^5s^2^ configurations, respectively. Electron–ion interactions were represented by ultrasoft pseudopotentials [31]. The numerical accuracy of the calculations was ensured by employing a plane-wave basis set truncated at 450 eV. Brillouin-zone sampling for the primitive cells of Mg and Al_3_Y was carried out using Monkhorst–Pack meshes of 9 × 9 × 9 and 7 × 7 × 9, respectively. Geometry optimizations were performed via the Broyden–Fletcher–Goldfarb–Shanno algorithm [32], allowing full relaxation of both atomic positions and lattice vectors until the total energy reached a minimum. Exchange–correlation effects were described using the generalized gradient approximation in the form of the Perdew–Burke–Ernzerhof revised for solids (PBEsol) functional, which is known to yield reliable structural parameters for metallic and intermetallic systems, and a Hubbard U correction of 2 eV was applied to the Y 4d states [33,34]. The self-consistent convergence criteria were defined as 1 × 10^−6^ eV for the total energy, 0.01 eV/Å for the maximum residual force, and 0.001 Å for atomic displacements.

Based on lattice matching considerations, the Mg(0001) and Al_3_Y(0001) surfaces were identified as the most suitable crystallographic combination for interface construction. Informed by our previous convergence tests on slab thickness for Mg, Ti, Mg_2_Y, and Al_2_Y systems [35,36], both the Mg(0001) and Al_3_Y(0001) slabs were constructed using seven atomic layers in the present study to ensure sufficient structural stability and interfacial convergence. For all slab calculations, a vacuum spacing of 15 Å was introduced perpendicular to the surface plane to suppress artificial interactions between periodically repeated images. Since only a single surface termination of the Al_3_Y slab was considered in our calculations, no dipole correction was needed. We further investigated the effect of various alloying elements (Si, Ca, Ti, Mn, Cu, Zn, Zr, and Sn) at the interface. Notably, Mn is magnetic, and its spin polarization was explicitly considered in all relevant calculations. Reciprocal-space sampling for surface calculations employed k-point meshes of 9 × 9 × 1 for Mg(0001) and 7 × 7 × 1 for the Al_3_Y(0001) surfaces. Based on these surface models, Mg(0001)/Al_3_Y(0001) interface supercells were subsequently constructed by combining a seven-layer Mg(0001) slab with an eleven-layer Al_3_Y(0001) slab. For the interfacial configurations, a reduced 3 × 3 × 1 k-point grid was adopted, while all other computational parameters were kept identical to those used in the bulk calculations to maintain internal consistency.

## 3. Results and Discussion

### 3.1. Bulk and Surface Properties

**Figure 1:**
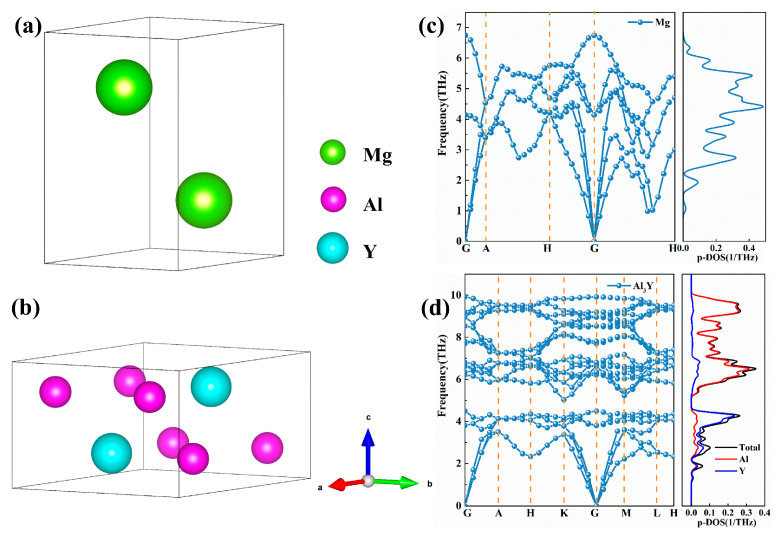
Crystal structure (**a**,**b**) and phonon spectrum (**c**,**d**) of Mg [36] and Al_3_Y compounds.

Both Mg and Al_3_Y crystallize in a hexagonal structure with the P6_3_/mmc space group, which provides inherent structural anisotropy along the basal plane and the c-axis, as shown in Figure 1a,b. Table 1 shows the lattice constants of Mg and Al_3_Y compounds. Notably, for magnesium, the initial lattice parameters were calculated as a = b = 3.17 Å and c = 5.14 Å, corresponding to a c/a ratio of 1.62, which increased to a = b = 3.18 Å and c = 5.32 Å after full relaxation, giving a c/a ratio of 1.67. Experimentally, Mg exhibits lattice constants of a = b = 3.209 Å and c = 5.211 Å [37]; using LDA calculations, the values are a = b = 3.159 Å and c = 5.073 Å, while GGA-PBE calculations yield a = b = 3.221 Å and c = 5.172 [38]. The small deviations between the GGA-PBEsol results and the experimental data indicate that the PBEsol functional yields an accurate description of the hexagonal Mg lattice, including both the basal plane and c-axis dimensions, thereby justifying its use in the present study. Similarly, Al_3_Y exhibits initial lattice parameters of a = b = 6.27 Å and c = 4.59 Å, and relaxed values of a = b = 6.28 Å and c = 4.60 Å, with both cases corresponding to a c/a ratio of 0.73. The increase in the c/a ratio of Mg after relaxation reflects an anisotropic elastic response of the hexagonal lattice under interfacial constraints, characterized by a slight expansion along the c direction and a concomitant contraction in the basal plane. In contrast, the nearly unchanged c/a ratio of Al_3_Y suggests a highly rigid and stable hexagonal framework, particularly along the c-axis. The comparable hexagonal symmetry and the small lattice mismatch between the basal planes of Mg and Al_3_Y are favorable for coherent interface formation. Specifically, the in-plane lattice constants of Mg (3.18 Å) and Al_3_Y (6.28 Å) suggest that a 2:1 lattice matching can be achieved with minimal strain, which is beneficial for interfacial stability and strong adhesion. The relaxation-induced expansion along the c-axis of Mg and slight adjustments in Al_3_Y further imply that both materials can accommodate interfacial strain without significant structural distortion, highlighting their potential compatibility in composite or alloyed systems. This lattice compatibility is expected to reduce misfit dislocations at the interface and promote favorable electronic interactions, which are critical for both mechanical stability and overall structural integrity in Mg/Al_3_Y composites.

To further verify the dynamical stability of Mg and Al_3_Y, phonon dispersion relations together with the corresponding phonon density of states (P-DOS) were calculated, as shown in Figure 1c,d. The results reveal that no imaginary frequencies are present throughout the entire Brillouin zone for either Mg or Al_3_Y, confirming that both crystal structures are dynamically stable at 0 K. For Mg, the phonon vibration frequencies are primarily distributed within the range of 0–7 THz, exhibiting a relatively narrow and concentrated spectrum. The corresponding P-DOS peak intensity does not exceed 0.45 (1/THz), which is characteristic of the fairly uniform atomic vibrations commonly observed in lightweight metallic systems. This behavior is consistent with the low atomic mass of Mg and its relatively weak bonding stiffness, and it correlates well with the smaller elastic moduli obtained from the mechanical property analysis. In contrast, the phonon spectrum of Al_3_Y displays pronounced element-resolved characteristics, with vibrational modes extending over a broader frequency range of 0–10 THz. In particular, the high-frequency region between 6 and 10 THz is dominated by contributions from Al atoms, whereas the low-frequency modes below 4 THz are primarily associated with the vibrations of Y atoms, with the overall P-DOS peak intensity remaining below 0.35 (1/THz). This distinct spectral separation can be attributed to the substantial difference in atomic mass and bonding stiffness between Al and Y, whereby the lighter Al atoms preferentially contribute to high-frequency vibrational modes, while the heavier Y atoms predominantly participate in low-frequency phonon behavior.

In the field of structural materials, mechanical performance plays a pivotal role in determining both service reliability and long-term durability. Under practical operating conditions, structural components are often subjected to complex mechanical loading, thermal fluctuations, and microstructural evolution, which may induce stress accumulation, phase transformations, or deformation incompatibilities. Insufficient mechanical robustness under such conditions can result in progressive damage, structural instability, or even catastrophic failure, thereby severely limiting the service lifetime and reliability of engineering components. For emerging lightweight alloys and advanced intermetallic or rare-earth-containing materials, achieving a balanced combination of mechanical strength, stability, and damage tolerance is therefore essential for their safe and efficient deployment in structural applications. The elastic constants and elastic modulus of Mg and Al_3_Y compounds are listed in Table 2. For HCP crystals, there are five independent elastic constants (C_11_, C_12_, C_13_, C_33_, and C_44_). Based on the calculated results in Table 2, the elastic constants C_ij_ of HCP Mg are C_11_ = 55.44, C_33_ = 71.16, C_44_ = 19.89, C_12_ = 30.73, and C_13_ = 23.87 GPa [35], while those of HCP Al_3_Y are C_11_ = 161.25, C_33_ = 186.57, C_44_ = 71.01, C_12_ = 65.33, and C_13_ = 24.15 GPa. All calculated elastic constants satisfy the mechanical stability criteria for HCP crystals, indicating that both Mg and Al_3_Y are mechanically stable [39,40]. Moreover, the calculated elastic constants show that Al_3_Y has significantly higher C_ij_ values than Mg, indicating a much stiffer HCP lattice. In particular, the larger C_11_ and C_33_ reflect greater resistance to axial deformation, while the higher C_44_, C_12_, and C_13_ indicate enhanced shear and in-plane rigidity, consistent with Al_3_Y’s role as a strengthening phase in Mg-based alloys. For magnesium, the computed bulk modulus (B), shear modulus (G), and Young’s modulus (E) are 37.60, 16.79, and 43.85 GPa, respectively. These values are in good agreement with experimental data, where B = 35.4 GPa and E = 43 GPa [38], indicating that the computational approach employed in this study can reliably reproduce the mechanical response of Mg. In contrast, Al_3_Y exhibits significantly higher elastic stiffness, with calculated values of B = 79.36 GPa, G = 62.35 GPa, and E = 148.23 GPa, reflecting its rigid intermetallic nature. The relatively soft Mg matrix can accommodate strain through elastic deformation, whereas the stiff Al_3_Y phase provides reinforcement, potentially enhancing the overall structural strength of Mg/Al_3_Y composites.

### 3.2. Properties of the Mg/Al3Y Interface

#### 3.2.1. Interfacial Configuration

**Figure 2:**
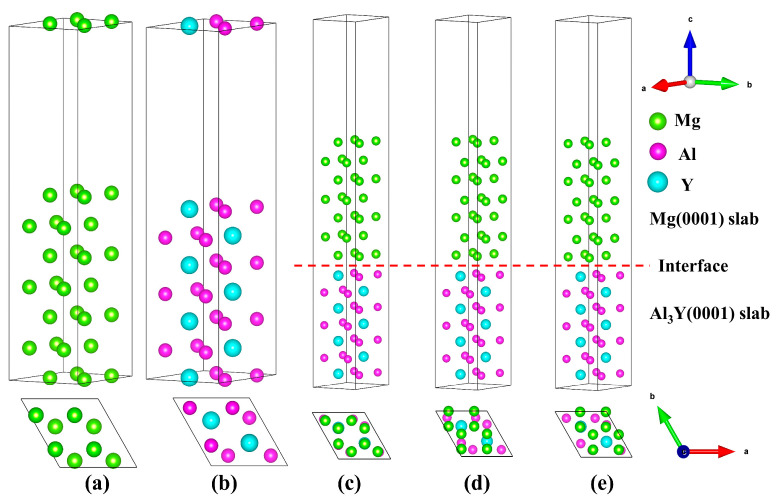
Surface and interface models. (**a**) Mg(0001) model, (**b**) Al_3_Y(0001) model, (**c**) Mg(0001)/Al_3_Y(0001)-OT configuration, (**d**) Mg(0001)/Al_3_Y(0001)-MT configuration, and (**e**) Mg(0001)/Al_3_Y(0001)-HCP configuration, and the red dashed line indicates the interface position.

Based on the crystallographic characteristics of Mg and Al_3_Y, the Al_3_Y crystal structure features Al and Y atoms occupying the Wyckoff positions 6 h (0.145, 0.290, 0.25) and 2d (1/3, 2/3, 3/4), respectively, while Mg atoms in the Mg crystal are located at the 2c (1/3, 2/3, 1/4) site. Such atomic site occupancies within the same hexagonal crystal system and space-group symmetry provide an essential structural basis for achieving atomically ordered stacking at the Mg(0001)/Al_3_Y(0001) interface. The atomic configurations of the seven-layer Mg(0001) and Al_3_Y(0001) surface slabs are illustrated in Figure 2a,b. Owing to the intrinsic crystallographic symmetry of Mg and Al_3_Y, only a single surface termination is present for both the Mg(0001) and Al_3_Y(0001) surfaces. Accordingly, as inferred from the lattice parameters listed in Table 1, a 2 × 2 in-plane supercell expansion of the Mg(0001) surface enables excellent lattice matching with the Al_3_Y(0001) surface, thereby substantially reducing the interfacial lattice mismatch. The lattice mismatch ratio at the interface can be quantified using the following expression:

(1) $$\xi =1-\frac{2\Omega }{A_{1}+A_{2}}$$

where ξ is the interface mismatch, Ω represent the interfacial area of the Mg(0001)/Al_3_Y(0001) interface, and *A*_1_ and *A*_2_ are the areas of the Mg(0001) surface and Al_3_Y(0001) surface, respectively. Based on the lattice parameters of Mg and Al_3_Y, *A*_1_ is defined as the effective in-plane lattice constant of the Mg(0001) surface after a 2 × 2 lateral supercell expansion, yielding a value of 6.36 Å, whereas *A*_2_ corresponds to the in-plane lattice constant of the Al_3_Y(0001) surface, which is 6.28 Å. Since both surfaces share an identical in-plane angle of 120°, no additional rotational alignment is required during interface construction, and only the lattice-parameter mismatch needs to be considered. Substituting these values into Equation (1) results in an interfacial lattice mismatch of approximately 1.27%. This value is well below the commonly accepted threshold of 5%, indicating that the Mg(0001)/(2 × 2)Al_3_Y(0001) interface exhibits excellent geometrical lattice compatibility and is therefore well suited for constructing a low-strain, semi-coherent interfacial model. From an atomic arrangement perspective, the hexagonal close-packed structure of the Mg(0001) surface exhibits a high degree of in-plane symmetry compatibility with the periodic distribution of Al–Y atomic layers on the Al_3_Y(0001) surface, which facilitates the establishment of strong interatomic interactions across the interface. Consequently, the Mg(0001)/(2 × 2)Al_3_Y(0001) interfacial configuration not only demonstrates excellent geometric lattice compatibility but also provides a robust and reliable structural model for subsequent investigations of interfacial adhesion strength, electronic structure, and mechanical response. The Mg(0001)/Al_3_Y(0001) interfacial configuration with different stacking configurations are illustrated in Figure 2c–e. In the “OT” configuration, the interfacial Mg atoms are positioned directly above the atoms in the topmost layer of the Al_3_Y slab. For the “MT” configuration, the Mg atoms at the interface are located above the midpoint of the nearest-neighbor atomic bonds in the first Al_3_Y layer. In contrast, in the “HCP” configuration, the interfacial Mg atoms are aligned above the atoms in the second atomic layer of Al_3_Y [41]. The interface is indicated by a red dashed line, with the Al_3_Y(0001) slab occupying the upper region and the Mg(0001) slab forming the lower region of the interface. Moreover, the red dashed line denotes the interfacial plane, with the region above the dashed line corresponding to the Mg(0001) slab and the region below representing the Al_3_Y(0001) slab.

In interfacial studies, the work of adhesion, W_ad_, is widely employed as a key parameter for quantitatively evaluating interfacial bonding strength. It is defined as the interfacial energy required, under thermodynamically reversible conditions, to separate an intact interface into two mutually independent free surfaces, normalized by the interfacial area. Accordingly, a higher W_ad_ value signifies stronger atomic interactions and more stable bonding characteristics at the interface. Based on this definition, the W_ad_ of the Mg(0001)/Al_3_Y(0001) interface is calculated and predicted in the present work [42,43]:

(2) $$W_{\mathrm{ad}}=\left(E_{Mg}^{slab}+E_{Al_{3}Y}^{slab}-E_{Mg/Al_{3}Y}^{\mathrm{int}erface}\right)/A_{i}$$

**Figure 3:**
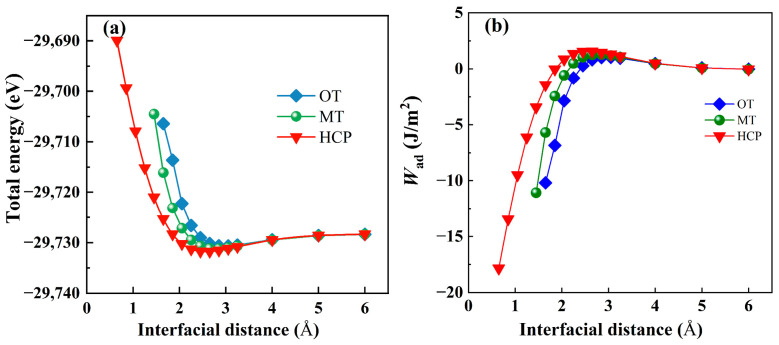
Total energy (**a**) and the work of adhesion (**b**) of Mg(0001)/Al_3_Y(0001) interface.

Here, $E_{Mg}^{slab}$, $E_{Al_{3}Y}^{slab}$, and $E_{Mg/Al_{3}Y}^{\mathrm{int}erface}$ are the total energies of the Mg(0001) slab, Al_3_Y(0001) slab, and Mg(0001)/Al_3_Y(0001) interface, and *A*_i_ is the area of the corresponding interface. Furthermore, Figure 3 illustrates the dependence of the total interfacial energy and the work of adhesion on the interfacial separation. With increasing separation from 1 to 6 Å, the total energy decreases rapidly at short distances and then gradually converges to a constant value. In contrast, the work of adhesion increases initially and subsequently reaches a plateau. From these trends, the equilibrium interfacial separation is determined to be approximately 2.6 Å.

#### 3.2.2. Interfacial Stabilities and Electronic Structure

**Figure 4:**
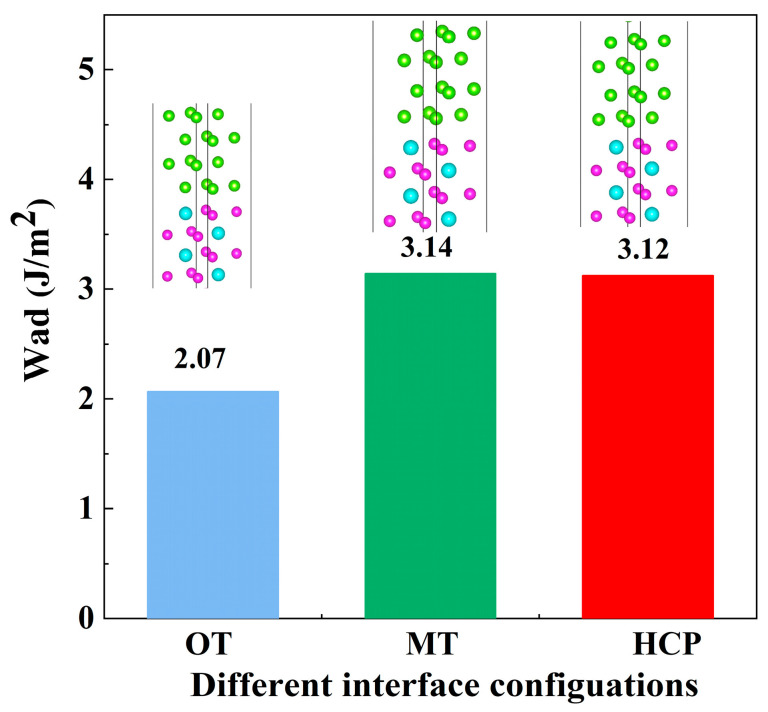
Work of adhesion of Mg(0001)/Al_3_Y(0001) interface.

The calculated W_ad_ for the Mg(0001)/Al_3_Y(0001) interface is presented in Figure 4 and listed in Table 3. The W_ad_ values for the OT, MT, and HCP configurations are 2.07, 3.14, and 3.12 J/m^2^, respectively. It can be clearly seen from Table 3 that the calculated W_ad_ shows that the Mg(0001)/Al_3_Y(0001) interface has the strongest bonding among the studied systems. The Mg(0001)/Al_3_Y(0001) interface with OT, MT, and HCP configurations exhibits W_ad_ values of 2.07, 3.14, and 3.12 J/m^2^, higher than Mg/Al_2_Y (1.19–1.68 J/m^2^) and most Mg/Mg_2_Y interfaces (0.43–2.38 J/m^2^). Only the Y-site MT/HCP Mg/Mg_2_Y interfaces approach Mg/Al_3_Y with OT configuration values but remain lower than Mg/Al_3_Y with MT/HCP configurations. This indicates that Mg/Al_3_Y, especially MT and HCP, benefits from stronger orbital hybridization and charge transfer, while Mg/Al_2_Y and Mg/Mg_2_Y generally show weaker bonding with smaller stacking-dependent differences, except for sites like Mg1-MT in Mg/Mg_2_Y, which is notably low. A comparison of these values indicates that both the MT and HCP configurations exhibit significantly higher W_ad_ than the OT configuration, with their values being very close to each other, suggesting stronger interfacial bonding in these arrangements. In contrast, the OT configuration shows the lowest W_ad_, implying relatively weaker atomic interactions and reduced interfacial stability. These differences can be primarily attributed to the variations in the local atomic coordination and bonding characteristics arising from different stacking arrangements. In the MT and HCP configurations, the interfacial Mg atoms achieve more favorable geometric matching and enhanced atomic contact with the Al_3_Y substrate, thereby strengthening chemical bonding and atomic interactions at the interface. By contrast, in the OT configuration, the interfacial atoms occupy top sites, resulting in stronger interatomic repulsion that hinders the formation of robust interfacial bonds. Accordingly, based on the magnitude of W_ad_, the MT and HCP configurations can be considered thermodynamically more stable, with the MT configuration exhibiting a slight advantage. This provides a solid basis for the subsequent analysis of interfacial stability and electronic structure.

**Figure 5:**
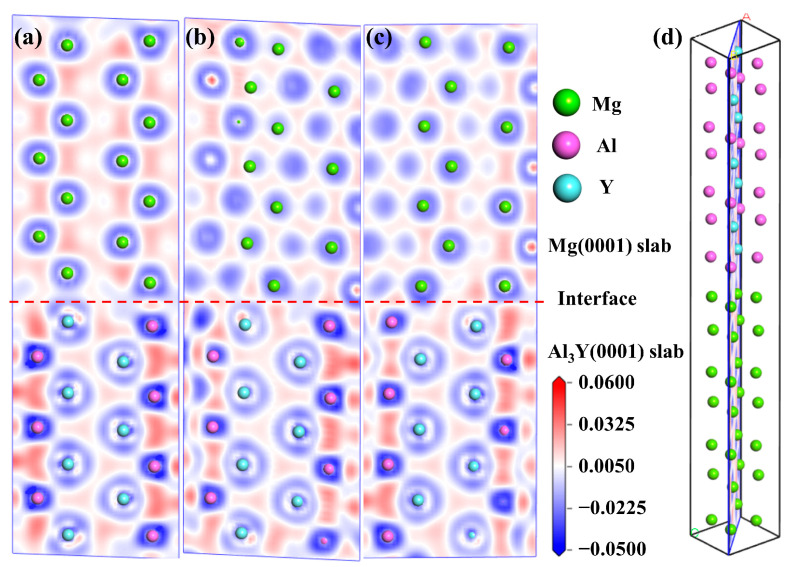
Charge density difference of Mg(0001)/Al_3_Y(0001) interface. (**a**) Mg(0001)/Al_3_Y(0001)-OT configuration, (**b**) Mg(0001)/Al_3_Y(0001)-MT configuration, (**c**) Mg(0001)/Al_3_Y(0001)-HCP configuration, and (**d**) schematic diagram of differential charge atomic cross-sections.

The differential charge density serves as an intuitive visualization tool for elucidating charge redistribution within a system. It is defined as the difference between the total charge density of the combined structure and the superposition of the charge densities of its constituent components in their isolated states. The corresponding expression is given as follows [44]:

(3) $$\Delta \rho =\rho_{crystal}-\sum \rho_{at}$$

where $\rho_{crystal}$ and $\rho_{at}$ are the valence electron densities of the compound and the reference-free atom, respectively. The charge density difference of Mg(0001)/Al_3_Y(0001) interface is shown in Figure 5. The differential charge density maps of Mg(0001)/Al_3_Y(0001) interface with OT, MT, and HCP configurations are shown in panels in Figure 5a–c, and the schematic diagram of differential charge atomic cross-sections is displayed in Figure 5d. Notably, the color scale represents electron accumulation (red region) and electron depletion (blue region). A pronounced charge redistribution is predominantly localized in the vicinity of the interface, whereas the inner layers of the Mg(0001) and Al_3_Y(0001) slabs largely retain their bulk-like electronic characteristics, indicating that the electronic interaction is highly confined to the interfacial region. Near the dashed line marking the interface, electron accumulation is clearly observed primarily in the bonding regions surrounding the interfacial Al and Y atoms, while electron depletion occurs around the Mg atoms and in other interstitial regions. This redistribution pattern signifies a substantial charge transfer from Mg to Al and Y atoms at the interface, which is consistent with the higher electronegativity of Al and Y compared with Mg. The accumulation of charge density in the bridging regions between Mg–Al and Mg–Y atomic pairs suggests the formation of directional interfacial bonds rather than purely metallic bonding. In particular, the more pronounced charge accumulation around Y atoms implies a stronger Mg–Y interaction, which contributes to enhanced interfacial cohesion. Furthermore, a comparison of Figure 5a–c reveals noticeable variations in both the spatial distribution and magnitude of charge accumulation, demonstrating that the interfacial stacking sequence plays a critical role in governing the extent of charge transfer and bonding strength. Configurations exhibiting more continuous and intense charge accumulation across the interface are therefore expected to possess higher interfacial adhesion, in good agreement with the calculated work of adhesion.

**Figure 6:**
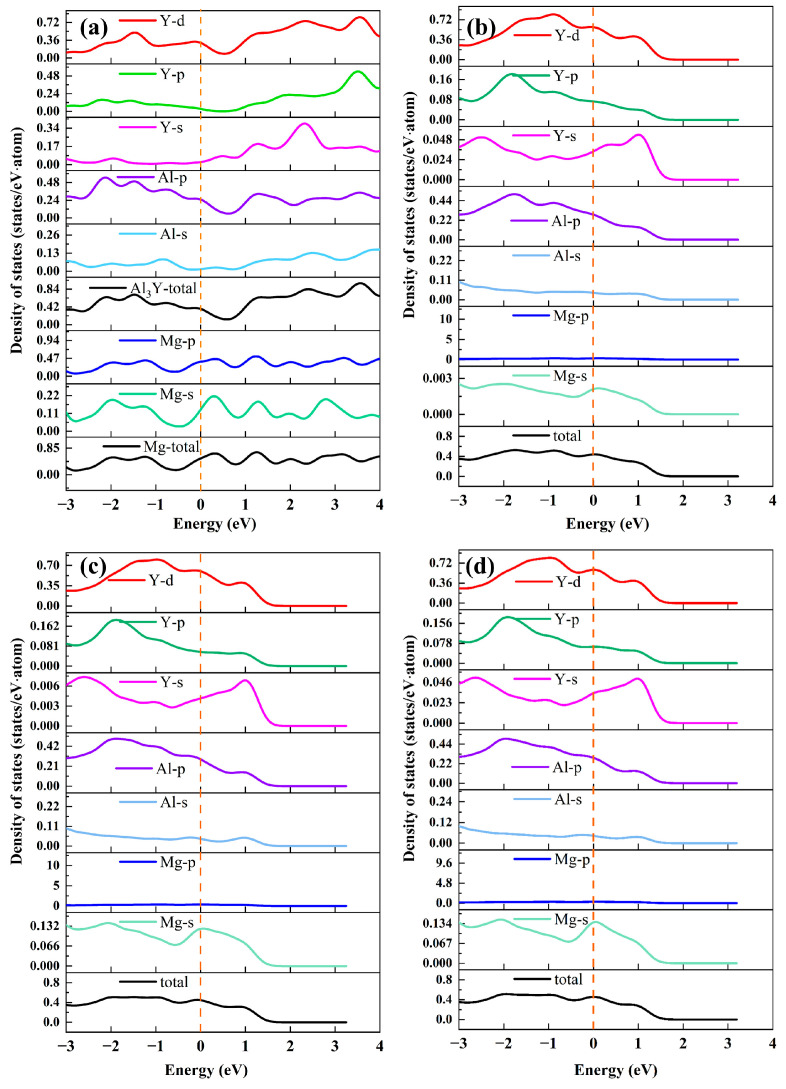
Density of states for bulk Mg, bulk Al_3_Y, and Mg(0001)/Al_3_Y(0001) interface. (**a**) Bulk Mg and Al_3_Y, (**b**) Mg(0001)/Al_3_Y(0001)-OT configuration, (**c**) Mg(0001)/Al_3_Y(0001)-MT configuration, and (**d**) Mg(0001)/Al_3_Y(0001)-HCP configuration, and the yellow dashed line indicates the Fermi level.

The total density of states (TDOS) represents the total number of available electronic states within a given energy interval (E to E + ΔE) and is obtained by integrating the electronic band states over all k points in the Brillouin zone. It thus reflects the overall energy distribution of electronic states in the material. The densities of states for bulk Mg, bulk Al_3_Y, and the Mg(0001)/Al_3_Y(0001) interface are shown in Figure 6. Generally, only the DOS changes near the Fermi level are considered; therefore, a DOS plot from –3 to 4 eV is presented. Figure 6a presents the projected density of states (PDOS) of the bulk Mg and Al_3_Y. It is observed that the states close to the Fermi level (EF) are predominantly contributed by Mg-s/p, Al-p, and Y-d orbitals. The presence of a finite density of states at the Fermi level indicates the pronounced metallic character of Mg and Al_3_Y. From the DOS in Figure 6b–d, it is apparent that all three interfacial structures maintain a nonzero density of states at EF (marked by the orange dashed line), confirming that the metallic character of the interfaces persists. This implies that the formation of the interface does not compromise the electronic conductivity of the system and provides favorable conditions for electronic interactions and charge transfer across the interface. The projected density of states (PDOS) further reveals that the electronic states near E_F_ are mainly contributed by Mg-s, Mg-p, Al-s, and Al-p, as well as Y-s, Y-p, and Y-d orbitals. Notably, the Y-d states display prominent features in the vicinity of E_F_ and show substantial overlap with the Al-p and Mg-p states within the same energy range, indicating strong orbital hybridization at the interface. In conjunction with the differential charge density analysis, electron depletion is observed around interfacial Mg atoms, whereas pronounced electron accumulation occurs near Al and Y atoms and in the interstitial regions between them. This charge redistribution is highly consistent with the energy overlap between Mg-p and Mg-p/Al-p states identified in the PDOS, confirming that interfacial bonding is accompanied by charge transfer from Mg to Al/Y atoms. Such charge transfer, together with orbital hybridization, gives rise to relatively directional interfacial chemical bonds rather than purely metallic bonding. Comparing the DOS of the interfacial structures in Figure 6b–d with the bulk DOS, it is evident that the interface formation induces modifications near the Fermi level, reflecting local electronic redistribution. Certain orbitals, particularly Mg-p and Y-d, show enhanced contributions at the interface, highlighting the increased role of specific elements in interfacial bonding. In some instances, splitting or reduction of peaks near EF indicates localized states or altered bonding characteristics. Furthermore, differences among the three interfacial configurations (OT, MT, HCP) illustrate how stacking or adsorption sites influence the DOS distribution. Notably, the MT configuration exhibits the strongest hybridization, corresponding to more pronounced interfacial charge accumulation and stronger Mg–Y interactions. This trend aligns well with the variation in the calculated Wad, confirming that enhanced orbital hybridization and charge transfer lead to stronger interfacial bonding.

#### 3.2.3. Interfacial Segregation and Adhesion Enhancement

**Figure 7:**
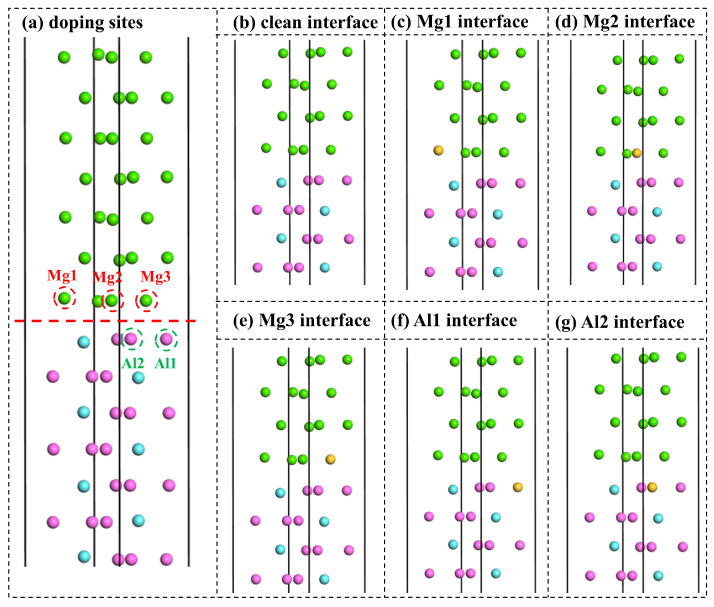
Mg(0001)/Al_3_Y(0001) interface doping models. (**a**) Doping sites, (**b**) clean interface, (**c**) Mg_1_ site, (**d**) Mg_2_ site, (**e**) Mg_3_ site, (**f**) Al_1_ site, and (**g**) Al_2_ site. The green, pink, and sky-blue spheres represent Mg, Al, and Y atoms, respectively, and the yellow balls represent doping atoms, including Si, Ca, Ti, Mn, Cu, Zn, Zr, and Sn.

The segregation behavior of doping elements at interfaces plays a decisive role in governing interfacial stability and bonding strength, originating from the unique low-coordination atomic environment and the complex electronic structure characteristic of interfacial regions. When doping elements can reduce the total energy of the system through charge redistribution, enhanced orbital hybridization, or effective relaxation of local strain at the interface, they tend to exhibit a pronounced segregation preference. Interfacial segregation not only reconstructs the local chemical composition but also markedly influences interfacial bonding characteristics, electronic structure evolution, and key properties such as the work of adhesion. Therefore, a systematic evaluation of the segregation energies of doping elements at different interfacial sites is of significant theoretical and practical importance for elucidating the microscopic mechanisms underlying interfacial stabilization and for guiding the design of high-performance alloy interfaces. On this basis, the Mg(0001)/Al_3_Y(0001) with MT configuration, which exhibits the highest interfacial stability, was selected to systematically investigate the effects of Si, Ca, Ti, Mn, Cu, Zn, Zr, and Sn doping elements on interfacial structure, adhesion behavior, and segregation characteristics. Interstitial doping or Y-site substitution was not considered, as the dopant elements studied (Si, Ca, Ti, Mn, Cu, Zn, Zr, and Sn) have considerably larger atomic radii than Y. Substitution at Y sites is therefore energetically unfavorable and likely to induce significant lattice distortion and local stress, which may destabilize the Al_3_Y structure and complicate the interpretation of interfacial properties. The Mg(0001)/Al_3_Y(0001) interface doping models are shown in Figure 7. Based on the crystal structural symmetry, the Mg1, Mg_2_, and Mg_3_ sites were defined on the Mg(0001) side, while the Al_1_ and Al_2_ sites were selected on the Al_3_Y(0001) side. The corresponding interfacial configurations after doping are illustrated in Figure 7c–g, in which yellow balls represent doping atoms, including Si, Ca, Ti, Mn, Cu, Zn, Zr, and Sn.

Furthermore, the W_ad_ of Mg(0001)/Al_3_Y(0001) after doping elements can be expressed as the following formulas [45,46]:

(4) $$W_{ad}=(E_{Mg}^{slab}+E_{Al_{3}Y-TM}^{slab}-E_{Mg/Al_{3}Y-TM}^{\mathrm{int}erface})/A_{i}$$

(5) $$W_{ad}=(E_{Mg-TM}^{slab}+E_{Al_{3}Y}^{slab}-E_{Mg-TM/Al_{3}Y}^{\mathrm{int}erface})/A_{i}$$

**Figure 8:**
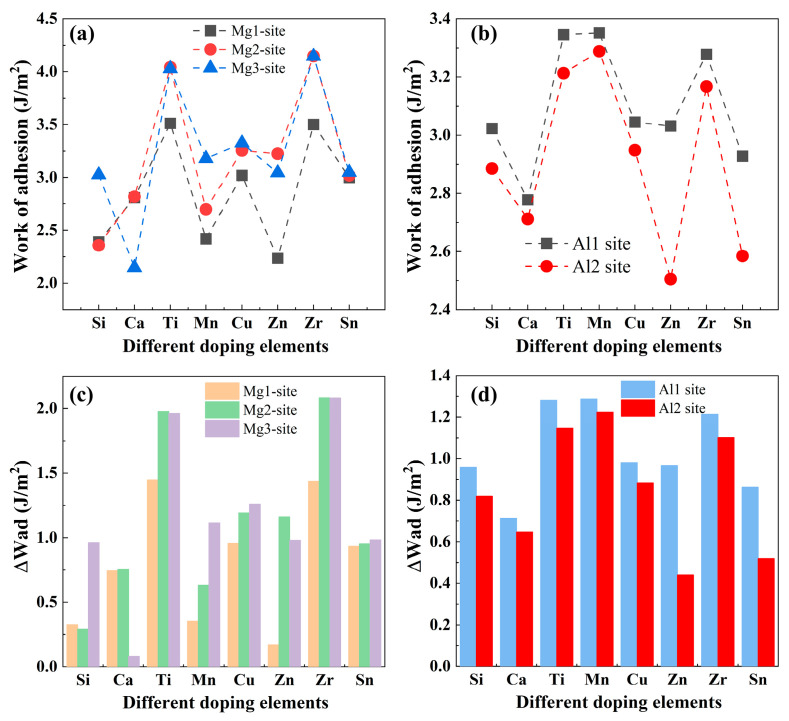
Work of adhesion and rate of change of work of adhesion for the doping Mg(0001)/Al_3_Y(0001) interface with different sites. (**a**,**c**) Mg sites and (**b**,**d**) Al sites.

Here, Equations (4) and (5) correspond to the models in which doping is introduced at the Al and Mg sites, respectively. In addition, $E_{Mg}^{slab}$, $E_{Mg-TM}^{slab}$,$E_{Al_{3}Y}^{slab}$, $E_{Al_{3}Y-TM}^{slab}$, $E_{Mg-TM/Al_{3}Y}^{\mathrm{int}erface}$, and $E_{Mg/Al_{3}Y-TM}^{\mathrm{int}erface}$ are the total energies of the Mg(0001) slab, doping Mg(0001) slab, Al_3_Y(0001) slab, doping Al_3_Y(0001) slab, Mg(0001)/Al_3_Y(0001) interface, and doping Mg(0001)/Al_3_Y(0001) interface. The calculated W_ad_ and rate of change of W_ad_ for the doping Mg(0001)/Al_3_Y(0001) interface with different sites are displayed in Figure 8. The data in Figure 8a,b clearly demonstrate that the effect of doping elements on the W_ad_ is strongly dependent on the interfacial site they occupy. Overall, Zr and Ti exhibit the most pronounced interfacial strengthening effect, with consistently higher Wad values than the other elements. In particular, when occupying the Mg_2_ and Mg_3_ sites, their Wad values exceed 4.0 J/m^2^, indicating that Zr and Ti preferentially form strong interfacial bonding on the Mg side, thereby markedly enhancing interfacial stability. In contrast, Mn, Cu, Sn, Si, and Zn yield Wad values mainly in the range of 2.8–3.3 J/m^2^, corresponding to a moderate improvement in interfacial adhesion; however, their strengthening effect is more sensitive to the specific interfacial site, with the Mg_2_/Mg_3_ sites generally being more favorable than the Al_1_/Al_2_ sites. By comparison, Ca exhibits relatively low Wad values at all interfacial sites, with a minimum of only approxiniamtely 2.1 J/m^2^, suggesting a limited contribution to interfacial adhesion enhancement. Taken together, these results indicate that Mg-side interfacial sites, particularly Mg_2_ and Mg_3_, are more effective in strengthening interfacial bonding, and that Zr and Ti are the most promising doping elements for maximizing the work of adhesion and interfacial stability, whereas the other elements are better suited as auxiliary doping additions for fine-tuning interfacial properties. As shown in Figure 8c,d, the W_ad_ exhibits markedly different variation-rate characteristics for different doping elements when they occupy distinct interfacial sites, indicating a pronounced element-dependent sensitivity of W_ad_ to site selection. For the Mg-side sites, Zr and Ti display a particularly high site sensitivity, and except for Ca and Zn, most doping elements show a higher W_ad_ variation rate at the Mg_3_ site than at the other Mg sites, suggesting that the Mg_3_ site is more effective in amplifying the interfacial strengthening effect induced by doping. Specifically, their effectiveness is primarily attributed to the larger atomic radii, which help relieve local stress, as well as stronger d-electron orbital hybridization, which enhances chemical interactions with surrounding atom [47]. On the Al-side sites, all elements exhibit higher Wad variation rates when occupying the Al_1_ site compared with the Al_2_ site, with Zn and Sn showing the largest variations among different interfacial sites, indicative of a strong site-selection effect. It should be noted that although the Wad of Ca varies with the interfacial site, its absolute values remain relatively low, implying a limited contribution to the enhancement of interfacial adhesion. Overall, the pronounced variations in W_ad_ are predominantly associated with the Mg-side interfacial sites, highlighting that an appropriate choice of doping elements and their preferred interfacial occupancy provides an effective strategy for tuning the work of adhesion and improving interfacial stability.

In addition, the heat of segregation of the doping Mg(0001)/Al_3_Y(0001) interface with different sites can predicted by the following expressions [48,49,50,51]:

(6) $$\Delta E_{seg}=\frac{1}{n}\left(E_{Mg/Al_{3}Y-TM}^{\mathrm{int}erface}-E_{Mg/Al_{3}Y}^{\mathrm{int}erface}+n\mu_{Al}-n\mu_{TM}\right)$$

(7) $$\Delta E_{seg}=\frac{1}{n}\left(E_{Mg-TM/Al_{3}Y}^{\mathrm{int}erface}-E_{Mg/Al_{3}Y}^{\mathrm{int}erface}+n\mu_{Mg}-n\mu_{TM}\right)$$

In Formulas (6) and (7), $E_{Mg/Al_{3}Y}^{\mathrm{int}erface}$ represents the total energy of the pure Mg/Al_3_Y interface, and $\mu_{Al}$, $\mu_{Mg}$, and $\mu_{TM}$ are the chemical potential of Al, Mg, and TM (TM = Si, Ca, Ti, Mn, Cu, Zn, Zr, and Sn), which are referenced to the energies of isolated single atoms; notably, spin polarization was explicitly considered when calculating the energy of the Mn atom. In addition, the chemical potential can be described as follows [52]:

(8) $$\mu_{TM}=\frac{E_{TM}^{bulk}}{n_{x}}$$

**Figure 9:**
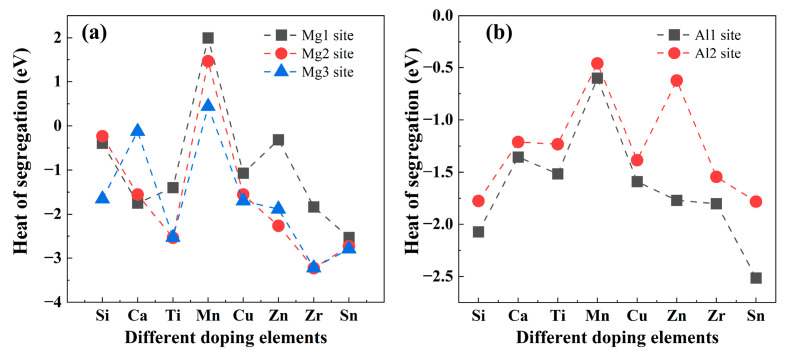
Heat of segregation of the doping Mg(0001)/Al_3_Y(0001) interface with different sites. (**a**) Mg sites and (**b**) Al sites.

In Equation (8), *n* denotes the number of doping atoms. In the present study, only a single doping atom is considered; therefore, *n* = 1. The calculated heat of segregation of the doping Mg(0001)/Al_3_Y(0001) interface with Mg sites and Al sites are shown in Figure 9. From the heat of segregation data shown in Figure 9, it is evident that different doping elements exhibit pronounced differences in their segregation behaviors at various interfacial sites. Overall, most doping elements display more negative segregation energies at the Mg-side sites, particularly at the Mg_2_ and Mg_3_ positions, indicating a stronger thermodynamic driving force for segregation toward the Mg-side interface, whereas the segregation tendency at the Al-side sites (Al_1_ and Al_2_) is comparatively weaker. Among the elements considered, Zr, Sn, and Ti exhibit the most negative segregation energies at the Mg_2_/Mg_3_ sites, suggesting the strongest propensity for interfacial segregation and implying their tendency to accumulate at the interface and form thermodynamically stable configurations. In contrast, Mn shows positive segregation energies at all Mg-side sites, indicating that Mn is unlikely to segregate to the interface; a similar trend is also observed at the Al_1_ site. With respect to the Al-side sites, all doping elements exhibit more negative segregation energies at the Al_1_ site than at the Al_2_ site, indicating a preferential segregation to the Al_1_ position, with Sn showing a particularly pronounced tendency. By comparison, Ca presents relatively small absolute values of segregation energy across all interfacial sites, approaching zero at the Mg_3_ site, which suggests a limited capability for interfacial segregation. Taken together, the Mg-side interfacial sites are thermodynamically more favorable for doping element segregation. Therefore, the rational selection of doping elements and their preferred interfacial occupancy is crucial for tailoring interfacial composition distributions and enhancing interfacial stability.

## 4. Conclusions

In this work, a systematic first-principles investigation was performed to elucidate the bulk properties of Mg and Al_3_Y and the interfacial structure–property relationships of the Mg(0001)/Al_3_Y(0001) system, with particular emphasis on interfacial stability, electronic bonding mechanisms, and doping element effects. The calculated lattice parameters, elastic constants, and phonon dispersion relations confirm that both Mg and Al_3_Y are dynamically stable and that the GGA-PBEsol functional provides a reliable description of their structural and mechanical properties. The similar hexagonal symmetry and small lattice mismatch (~1.27%) between Mg(0001) and Al_3_Y(0001) basal planes enable the construction of a low-strain semi-coherent interface, which is favorable for achieving strong interfacial adhesion in Mg/Al_3_Y composites. Among the three considered stacking configurations, the MT and HCP configurations exhibit significantly higher work of adhesion than the OT configuration, indicating that interfacial stability is highly sensitive to atomic stacking. Electronic structure analyses reveal pronounced charge transfer from Mg to Al and Y atoms at the interface, accompanied by strong orbital hybridization between Mg-p, Al-p, and Y-d states near the Fermi level, giving rise to partially directional interfacial bonds rather than purely metallic bonding. The MT configuration shows the strongest hybridization and charge accumulation, consistent with its highest work of adhesion and superior thermodynamic stability. Furthermore, the segregation behavior and strengthening effects of typical doping elements (Si, Ca, Ti, Mn, Cu, Zn, Zr, and Sn) were systematically evaluated. The results demonstrate that Mg-side interfacial sites, particularly Mg_2_ and Mg_3_, are thermodynamically more favorable for segregation and more effective in enhancing interfacial adhesion than Al-side sites. Among the elements considered, Zr and Ti exhibit the most negative segregation energies and the most pronounced improvement in work of adhesion, identifying them as highly effective elements for interfacial strengthening, whereas Mn and Ca show limited segregation tendency and adhesion enhancement. Overall, this study provides fundamental atomic-scale insights into interfacial stabilization mechanisms and offers clear theoretical guidance for doping element selection and interface engineering in high-performance Mg-based composite systems.

## Figures and Tables

**Table 1:** Lattice constants of Mg and Al_3_Y compounds.

Species	Method	a (Å)	c (Å)	c/a
Mg	GGA + PBEsol	3.18	5.32	1.67
	Before relax	3.17	5.14	1.62
	Exp. [37]	3.209	5.211	
	GGA + PBE [38]	3.221	5.172	
	LDA [38]	3.159	5.073	
Al_3_Y	GGA + PBEsol (this work)	6.28	4.60	0.73
	Before relax	6.27	4.59	0.73

**Table 2:** Elastic constants and elastic modulus of Mg and Al_3_Y compounds; the unit is GPa.

Species	Method	C_11_	C_33_	C_44_	C_12_	C_13_	B	G	E
Mg	GGA + PBEsol	55.44	71.16	19.89	30.73	23.87	37.60	16.79	43.85
	Exp. [37]						35.4		
	GGA + PBE [38]						35.6		
	LDA [38]						43.9		
Al_3_Y	GGA + PBEsol	161.25	186.57	71.01	65.33	24.15	79.36	62.35	148.23

**Table 3:** The work of adhesion of the Mg(0001)/Al_3_Y(0001) interface, together with a comparison with the adhesion work of other interfaces.

Species	Wad (J/m^2^)	Reference
Mg/Al_3_Y-OT	2.07	This work
Mg/Al_3_Y-MT	3.14	This work
Mg/Al_3_Y-HCP	3.12	This work
Mg/Al_2_Y-Al-OT	1.49	[35]
Mg/Al_2_Y-Al-MT	1.63	[35]
Mg/Al_2_Y-Al-HCP	1.56	[35]
Mg/Al_2_Y-Y-OT	1.19	[35]
Mg/Al_2_Y-Y-MT	1.54	[35]
Mg/Al_2_Y-Y-HCP	1.68	[35]
Mg/Mg_2_Y-Mg1-OT	0.94	[36]
Mg/Mg_2_Y-Mg1-MT	0.43	[36]
Mg/Mg_2_Y-Mg1-HCP	1.99	[36]
Mg/Mg_2_Y-Mg2-OT	1.80	[36]
Mg/Mg_2_Y-Mg2-MT	1.84	[36]
Mg/Mg_2_Y-Mg2-HCP	1.82	[36]
Mg/Mg_2_Y-Y-OT	1.99	[36]
Mg/Mg_2_Y-Y-MT	2.36	[36]
Mg/Mg_2_Y-Y-HCP	2.38	[36]

## Data Availability

The original contributions presented in this study are included in the article. Further inquiries can be directed to the corresponding author.
